# Stress and Strain Provide Positional and Directional Cues in Development

**DOI:** 10.1371/journal.pcbi.1003410

**Published:** 2014-01-09

**Authors:** Behruz Bozorg, Pawel Krupinski, Henrik Jönsson

**Affiliations:** 1 Computational Biology & Biological Physics, Lund University, Lund, Sweden; 2 Sainsbury Laboratory, Cambridge University, Cambridge, United Kingdom; University of Chicago, United States of America

## Abstract

The morphogenesis of organs necessarily involves mechanical interactions and changes in mechanical properties of a tissue. A long standing question is how such changes are directed on a cellular scale while being coordinated at a tissular scale. Growing evidence suggests that mechanical cues are participating in the control of growth and morphogenesis during development. We introduce a mechanical model that represents the deposition of cellulose fibers in primary plant walls. In the model both the degree of material anisotropy and the anisotropy direction are regulated by stress anisotropy. We show that the finite element shell model and the simpler triangular biquadratic springs approach provide equally adequate descriptions of cell mechanics in tissue pressure simulations of the epidermis. In a growing organ, where circumferentially organized fibers act as a main controller of longitudinal growth, we show that the fiber direction can be correlated with both the maximal stress direction and the direction orthogonal to the maximal strain direction. However, when dynamic updates of the fiber direction are introduced, the mechanical stress provides a robust directional cue for the circumferential organization of the fibers, whereas the orthogonal to maximal strain model leads to an unstable situation where the fibers reorient longitudinally. Our investigation of the more complex shape and growth patterns in the shoot apical meristem where new organs are initiated shows that a stress based feedback on fiber directions is capable of reproducing the main features of in vivo cellulose fiber directions, deformations and material properties in different regions of the shoot. In particular, we show that this purely mechanical model can create radially distinct regions such that cells expand slowly and isotropically in the central zone while cells at the periphery expand more quickly and in the radial direction, which is a well established growth pattern in the meristem.

## Author Summary

Development and morphogenesis of tissues are dependent on a coordination between cell differentiation, proliferation and growth. Plants, which lack cell migration, control directional growth of tissues by adjusting cellulose fiber directions so forming the organ shapes. It has recently been shown that mechanical cues can guide these fibers. We developed detailed mechanical models to investigate how fiber directions may be responding to mechanical cues and what consequences this may have for positional and directional growth patterns. We show that a model in which fibers align to maximal stress directions spontaneously generates a radial zonation in the shoot, recapitulating the slowly growing center and more rapidly growing peripheral region previously observed in the meristem. These radial patterns emerging from mechanics are in striking correspondence to the expression patterns of the genes important for stem cell maintenance, which attain similar radial domains. We also show that the stress model can robustly define anisotropically growing organs, which emphasizes the potential importance of stress in generating correct organ shapes in plants.

## Introduction

Mechanical forces are integral part of any living system and recent data is confirming their importance as signaling cues in animal and plant development [1]–[3]. This may be especially important for plants which have to sustain large loads while executing a developmental program that is optimal in their habitat [4]. Due to the lack of cell migration, plants must change the mechanical properties of their tissues on the cellular scale in order to facilitate directional growth of organs.

The mechanical properties of plant tissue can be linked down to the properties of cell walls. The walls are composed of a network of cellulose microfibers interconnected by polysaccharides and xyloglucans [5]–[7]. They constitute the structurally strong element of plant tissue providing support against turgor pressure and internal tension. From a mechanical point of view, the walls can be considered to be thin visco-elastic elements.

The epidermis of plant tissue is thought to play a special role in morphogenesis [8], [9]. It is generally more mechanically stiff than internal tissues, which suggest a ‘tissue pressure’ model where tensional forces in the epidermis are generated by the pressure and growth of the internal cells [8]. Under the action of hormones or enzymes the epidermis can experience substantial changes in its mechanical properties [10]–[12], which is determinant in the outgrowth of plant organs. The prevailing idea of how an isotropic tissue pressure generates anisotropic growth has to do with the anisotropy of plant material. The cellulose microfibers, which have been shown to have highly organized directional patterns in the epidermis [13], [14], restrict the elastic expansion of a tissue in the direction parallel to them. The organization of the wall fibers is regulated by cells via the deposition of cortical microtubules [15]. This fact has been exploited by experiments which often use microtubule direction as a proxy for fiber direction. While directional fibers can translate the isotropic forces into specific strain directions, additional mechanisms for long-term plastic anisotropic growth are also needed. The data suggests that such growth is the result of a molecular break and slip behavior with new material constantly being added to the walls [16], [17], where plastic growth is triggered by the stresses in the wall exceeding a yield threshold. When anisotropic material is generated by adding strong fibers, the picture becomes more complex, and the idea for how the growth proceeds is that weaker molecules connecting the fibers break and allow for extension in the direction perpendicular to the fibers [17]. While simple models of plant growth have been developed, a model for plant tissues that is compatible with the stress-based growth and anisotropic cell wall material has not been defined [18]–[20].

The composition of the plant cell wall is controlled by the genetic program of the cell which must allow for a large degree of adaptivity for the whole plant, the existence of specialized tissue types and the wealth of plant forms. However, as recent evidence [3], [21] and previous ideas [22] suggest, it is likely that reciprocal signaling, linking mechanical states of the tissues and cell walls to biochemical processes takes place too, connecting growth rate and direction with mechanical properties of the plant tissue in a feedback loop. Molecular details of the mechanism of such two way relations between mechanics and cell functions are still elusive and require further investigation [7]. In particular, the organization of the cellulose fibers, which leads to a directional growth, may be determined by several cues. One suggestion is that the fibers align orthogonally to the maximal strain direction. This has been proposed for anisotropically growing tissues [23], [24]. A more recent suggestion is that the fibers align in the maximal stress direction [25], which is supported by the fiber patterns observed in the plant meristem [3], [26]. For isotropic mechanical materials the two ideas would be easy to discriminate between, because then maximal principal strain and stress point in the same direction. However, for mechanically anisotropic plant walls, maximal strain and stress may very well be orthogonal and it may not be easy to discern between the two rules of fiber alignment. The situation is complicated further by the fact that a change in the fiber direction will lead to a change in stresses and strains resulting from the same external load. This complex feedback loop makes it difficult to predict *a priori* whether either stress or strain directions can act as stable inputs for shape generation, even if these directions are easily predicted given the material anisotropy. The intricate dynamics of fiber alignment resulting from such feedbacks has yet to be investigated in detail.

Mechanical strains and stresses in tissues are not easy to measure, so there is a need for reliable mechanical models of biological materials that can quantitatively predict both magnitude and direction of the strains and stresses. Using such models, after prescribing the material properties and loading forces, one can accurately describe the mechanical response of the tissue and further test different scenarios for how the mechanical response is coupled to biochemical signals. There exists a large variety of finite element or particle based methods which can be applied to modeling mechanical responses of materials [27]. These methods, however, are usually quite computationally intensive and large scale models of biological cells are not always feasible within them. In addition these methods have not been designed or optimized to cope with the dynamic complexity of biological materials and the growth of tissues, which require rapid changes to the model's cellular topology and material composition.

**Figure 1:**
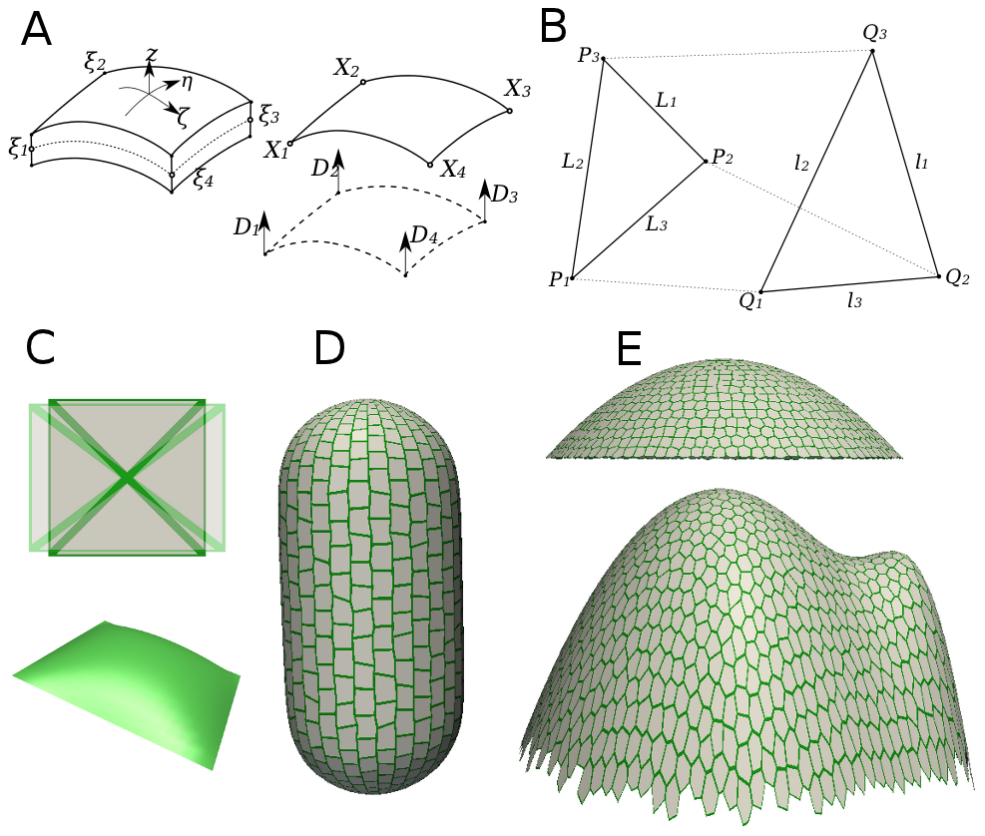
Mechanical models and templates. (A) Geometry of a quadrilateral shell element for the finite element method. The thin three-dimensional surface is parametrized by a two-dimensional shell with implicit thickness and set of director vectors *D* (Text S1). (B) An element used in the triangular biquadratic spring model. 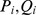 and 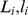 represent positions and edge lengths in resting and deformed state, respectively. The strain tensor can be expressed in terms of edges of the element in resting and deformed states. (C) The quadrilateral patch used for comparing triangular biquadratic springs and finite element shell models. (D, E) Different templates representing selected plant-like geometries used in tissue pressure simulations.

Given the geometry of a plant cell wall, where its thickness is often more than an order of magnitude smaller than its planar extension, finite element method (FEM) shell models provide an adequate description since they are specifically designed for thin curved surfaces and describe tensile and bending behavior (Figure 1A) [28], [29]. More recently, Triangular Biquadratic Spring (TRBS) models have been developed to describe two-dimensional elastic elements [30]. TRBS has the benefit of simplicity: this class of model describes mechanical responses using just the resting and current lengths of the triangular edges ({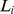}, {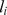} in Figure 1B). The TRBS implementation has been shown to accurately represent continuum properties of mechanics [30]. However, since in TRBS the bending energy is disregarded, it is not obvious that such models provide a good description of plant walls that typically consists of curved structures.

In this paper we develop two implementations of a mechanical model for anisotropic plant wall material: a FEM shell implementation and a TRBS plate implementation. We compare the implementations both in in-plane loading simulations and in tissue pressure models of the plant epidermis, the latter leading to additional bending moments in shells (Figure 1C–E). We analyze the relation between maximal (first principal) stress and strain directions under different loading forces. We use the method to analyze different proposed mechanisms of coupling between mechanical cues and alignment of material anisotropy of cells, based on perception of either maximal stress direction (MSD) or the direction orthogonal to maximal strain (OsD). We apply the models to different geometries representing different tissues in plants in order to evaluate their potential for explaining cellulose fibers patterns and growth patterns observed in epidermal plant tissues (Figure 1D–E).

## Results

### Shell Finite Elements and Triangular Biquadratic Springs offer an adequate description of anisotropic plant wall material

One of our goals was to establish an efficient computational method and a sufficiently accurate material model that can be used to simulate the behavior of plant walls. In particular, we aimed to investigate whether a Triangular Biquadratic Spring method can provide a reliable description, given that it is a two-dimensional representation and that it does not explicitly include any bending resistance. To do this we developed a TRBS method and compared the results with a shell-based finite element method (Methods and Text S1).

To describe the anisotropic wall material, we used a hyperelastic strain energy density formalism applicable to large strain deformations (Text S1). For the isotropic wall material we used a St. Venant-Kirchoff description [3], [30], and developed an anisotropic material model penalizing extension in a defined fiber direction (Equations 1,3 and Text S1).

**Figure 2:**
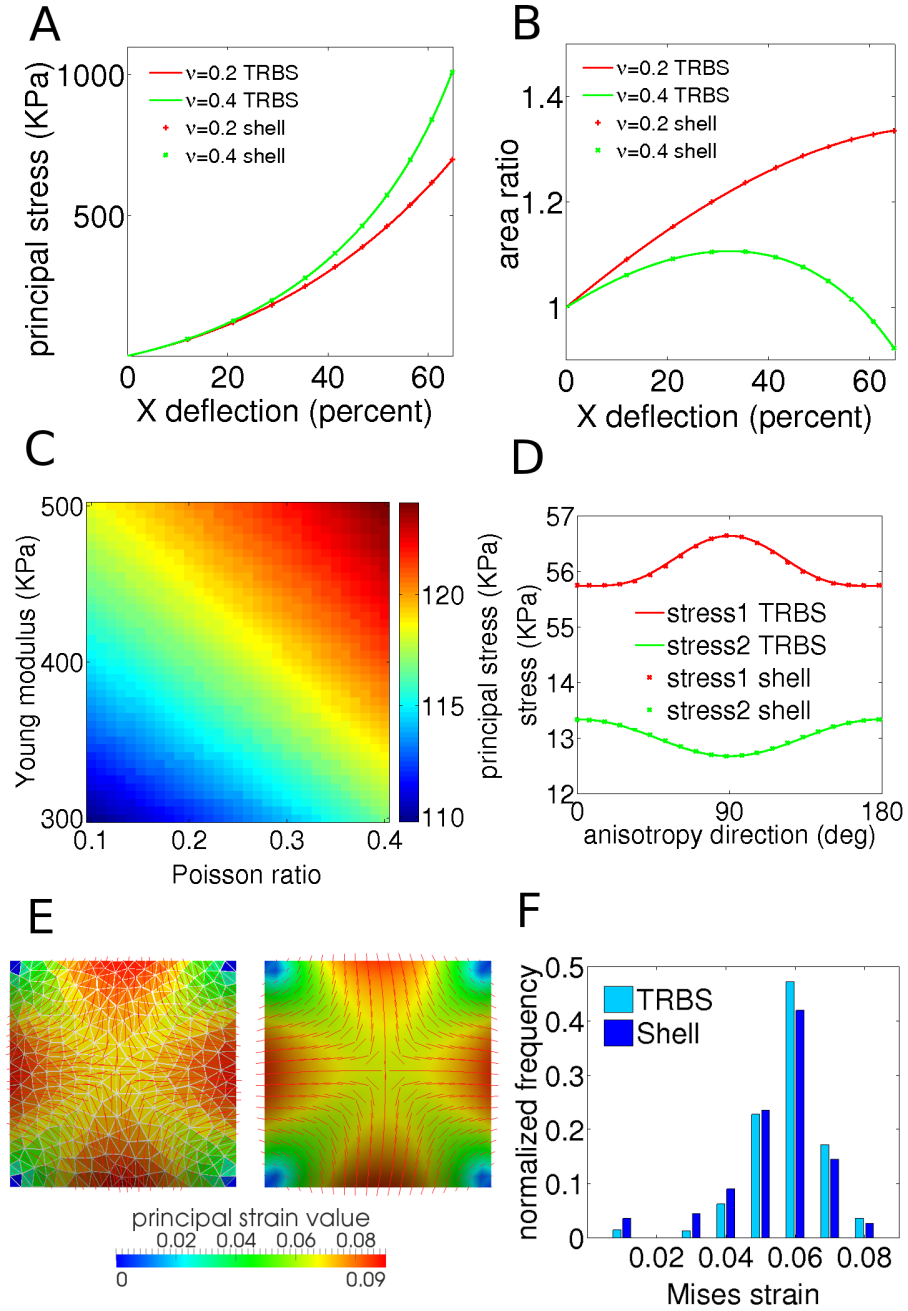
Comparing triangular biquadratic springs and finite element shell models. (A, B) Uniaxial stretching test on a quadrilateral patch shows prefect agreement within numerical accuracy between both methods for principal stress and area ratio versus deflection of top right corner of the quad. Isotropic material (Young modulus = 400 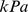, Poisson ratio = 0.2 and 0.4, thickness = 0.01 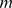, size = 1 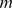, force = 8 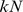). (A) Principal stress. (B) Area ratio. (C) Principal stress value for isotropically loaded patch with 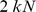 force for the same patch using TRBS method where Young modulus and Poisson ratio were varied. The difference between principal stress value in TRBS method and integrated principal stress over thickness in FEM shell model is less than 0.1% (Figure S1A). (D) First and second principal stress values for the same patch of anisotropic material with transverse and longitudinal Young modulus of 400 and 800 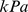 respectively and Poisson ratio of 0.2, under 0.8 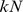 and 0.2 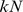 anisotropic loading force. The anisotropy direction was varied between 0 deg (maximal force direction) and 180 deg. (E, F) Bending test results from pressurizing a patch of elements. (E) Principal stress direction and principal strain value for TRBS (left) and shell (right). The material is isotropic with Young modulus 400 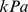 and Poisson ratio 0.2. Number of elements is 400 and 250 for shells and TRBS, respectively. (F) Distribution of equivalent Mises strain value over elements. TRBS elements show slightly higher strain values because of the lack of bending energy. Average equivalent Mises strain over elements: 0.0527 and 0.0492 for TRBS and shell, respectively.

First, we tested a mechanically isotropic square patch of elements under different loading conditions and for different material properties (Figure 1C). When we applied uniaxial tensions, the stress-strain relations completely agreed between the methods (Figure 2A–B). Further, the two methods agreed for a wide range of Young moduli and Poisson ratios under isotropic loading forces with a difference of less than 0.1 percent between them (Figure 2C, Figure S1A). Note that the principal stress value is a monotonically increasing function of not only the Young modulus but also of the Poisson ratio for this mechanical model (Figure 2B). We extended the uniaxial tension tests into a large deformation regime to demonstrate the well known deficiency of the St. Venant-Kirchoff material model, where uniaxial loading forces can result in infinite stresses and zero volume at finite strains [31]. We found that this deficiency appears especially when the Poisson ratio is high (Figure 2A–B). In simulations of plant tissues, we do not expect strains to exceed several percent, which corresponds to the typical values 5–10% encountered in experiments [32], and as such the model provides an appropriate description of plant wall material.

Next, we analyzed the response of the anisotropic material model for the square patch of elements under biaxial loading forces. Under isotropic loading forces an increased degree of material anisotropy led to an increased difference between the magnitude of principal stresses (Figure S1B), and the maximal stress and strain directions were perpendicular to the fiber direction. Under anisotropic loading forces, the response depended on the angle between the maximal force direction and the direction of the axis of material anisotropy (Figure 2D). When the material anisotropy direction coincided with the direction of the maximum loading force, the maximal principal stress value was lower than when those directions were perpendicular. This could have profound implications for plant wall mechanics. Since stresses trigger inelastic responses and breakage of brittle components of a material [33], a plant cell's ability to control the amount of stress in the tissue by adjusting its anisotropy could be a way of directing growth given the stress magnitude's relation to the yield stress of the wall material [5].

To assess the importance of the lack of bending resistance in the TRBS method we compared principal stress pattern, principal strain value and deformation with the FEM shell method for a pressurized quadrilateral plate (Figure 1C, 2E), different plant-like geometries (Figure 1E, Figure S1C–D), and a saddle-like plate (Figure S1E).

The results showed good agreement between the two methods for the pressurized quadrilateral plate suggesting that the deformation in our tissue pressure model is dominated by tensile and not bending stress (Figure 2E), although we found small quantitative differences. For example, the normalized distribution of equivalent von Mises strain for the TRBS method had a slightly higher average (0.052 vs. 0.049) (Figure 2F) probably owing to the lack of bending energy at the junctions. The agreement held for most geometries tested (Figure S1C–D), with exceptions where compressive forces generated buckling (Figure S1E) –yet, even in such cases the qualitative pattern and distribution of stresses was in good agreement between both methods. The good agreement of the two methods indicates that tensile stresses dominate over bending moments, but also that although the TRBS approach does not explicitly account for bending energies at individual edges, the triangulated mesh structure may still incorporate a resistance towards bending via stretch and compression of the elements induced by bending.

In conclusion, we have shown that TRBS and shell finite element methods strongly agree when applied to models of anisotropic wall material in two dimensions for a wide range of values of material anisotropy and applied forces. Although quantitative differences appear, the methods also show strong agreement in the case where two-dimensional structures are pressurized into three-dimensions and where bending forces are induced. We also found that for an anisotropic material under anisotropic loading forces a complex relation between the direction of maximal load and the directions of the maximal strains and stresses appear, indicating that plant cells can control these variables if they are able to control cellulose fiber directions.

### Mechanical strain and stress are not equivalent signals in the presence of material anisotropy and loading anisotropy

**Figure 3:**
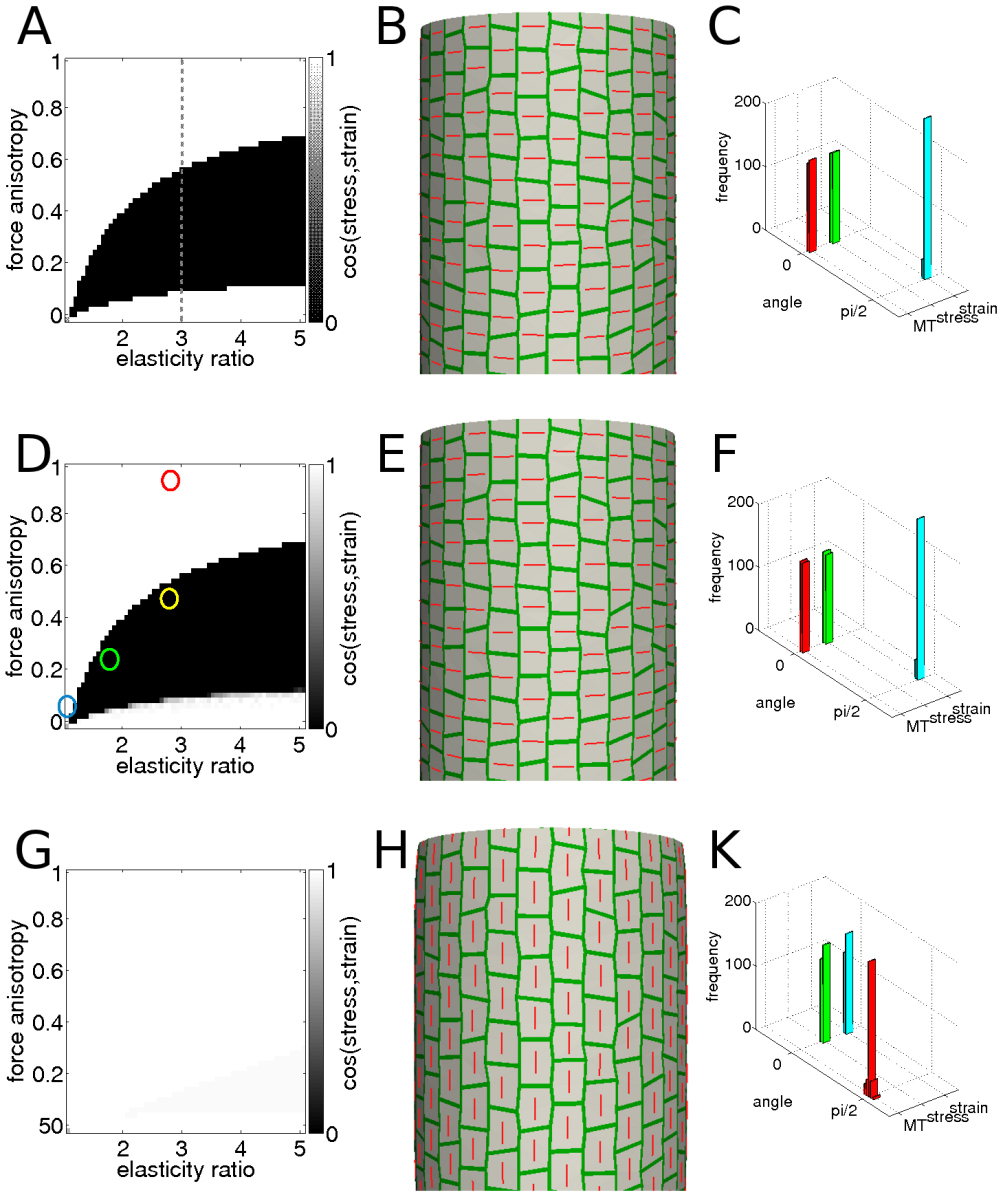
Comparison between stress and orthogonal strain based feedback models. The results of the three distinct relations between mechanical stress/strain and anisotropy of the material in different loading force situations are analyzed. The first row (A, B, and C) pertains to the predefined and static direction of material anisotropy. The second row (D, C and F) describes the results of stress feedback model and the third row (G, H and K) the orthogonal strain feedback model. The first column (A, D and G) presents the results of the simulation of anisotropic biaxial loading of a square patch from Figure 1C. For varied anisotropy of the loading force (vertical axis in the graphs) and the ratio of Young moduli along each of the load directions (horizontal axis in the graphs), the cosine of the angle between maximal stress and strain directions is plotted with a gray-scale map. Force anisotropy and elasticity ratio in A, D and G are calculated by 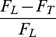 and 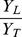, respectively. Force anisotropy 0 corresponds to isotropic loading and elasticity ratio 1 to an isotropic material. The gray dashed line in panel A and circles in panel D are discussed in the main text. The second column (B, E and H) shows the equilibrium state of fiber directions (red bars) in the cylindrical part of the tissue pressure model simulation for the template shown in the Figure 1D. The third column (C, F and K) pictures the distributions of the stress, strain and fiber directions in the cells with respect to the circumferential (horizontal) direction resulting from the tissue pressure model simulation. (A) For the fixed anisotropy direction (no feedback mechanism present) we observe distinct regions in the parameter space where maximal stress and strain directions are either mutually parallel (white) or perpendicular (black). (B) In the stem template simulations the anisotropy (fiber) direction is prealigned and set to circumferential. (C) This results in a maximal stress direction parallel to the fiber direction (circumferential) and maximal strain direction orthogonal to the fiber direction (longitudinal). (D) In the stress feedback model the identity of the regions of mutually parallel (black) or orthogonal (white) relation between the maxima stress and strain directions is maintained from the no-feedback case A. The yellow circle in D shows the approximate value for force and material anisotropy on the side of a cylinder where anisotropic curvature results in force anisotropy about 0.5. (E) In this model fibers are dynamically aligned in the direction of the maximal stress and the circumferential orientation of them arises spontaneously in the stem template simulation. (F) Similarly to the static case (first row) the maximal strain direction is perpendicular to the stress and fiber directions ie. longitudinal. (G) For the orthogonal strain feedback model the maximal stress and strain directions are always parallel in contrast to A and D. (H) In this case fibers are dynamically updated to match the direction orthogonal to maximal strain. This results in unstable initial circumferential alignment of fibers which realign in the longitudinal direction. (K) Both maximal stress and strain directions are perpendicular to the fiber directions ie. circumferential. The parameters used in the simulation with the pressurized template in Figure 1D were: thickness 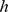 = 1 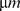, cell size 10 to 20 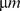, 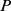 = 0.1 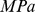, 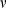 = 0.2, 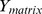 = 50 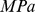, 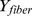 = 120 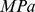, fiber model with 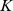 = 0.4 and 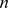 = 2, deformation is between 5% to 10% (B)6%, (E) 6%, (H) 10%).

To analyze the relation between stress and strain directions under different loading forces and for different fiber directions we first investigated a situation where the direction of maximal applied force coincided with the fiber direction in a simple square. The maximal stress direction always followed the maximal loading force direction. Depending on the degree of anisotropy of the applied force and the material properties, the resulting maximal direction of strain could be either parallel or perpendicular to the maximal stress direction marking distinct regions in the (force-anisotropy, material-anisotropy) parameter space (Figure 3A). As expected, for isotropic materials the maximal principal stress and strain directions both coincided with the maximal applied force direction. For anisotropic materials and anisotropic loads we obtained a region where maximal stress and strain directions can be perpendicular (black region in Figure 3A). The extension of this region depended on the Poisson ratio of the material (Figure S2A–C). Given a fixed material anisotropy (dashed line in Figure 3A), isotropic loading leads to parallel directions of the maximal stress and strain. A higher directional force can be resisted by the stronger component of the material leading to a maximal strain direction perpendicular to the maximal force direction. However, when the forces are highly anisotropic then they overcome the resistance of the stronger component of the material and the maximal strain follows the direction of the applied force.

This reveals that potential cellulose fiber orienting mechanisms based on the feedback from either stress or strain can behave differently from one another in some parts of a tissue while in other parts of the tissue they show the same behavior. We used the models to analyze the anisotropic growth of shapes resembling plant organs where the alignment of fibers in epidermal tissues is thought to guide growth. We simulated a cylindrically shaped tissue using the tissue pressure model and parameter values from experimental estimates [34]–[36] and recovered the expected stress about half in the longitudinal direction compared to the circumferential direction. We set the fiber direction to be circumferential to match observed microtubule directions in the epidermis of several plant tissues [3], [37]–[39]. This led to a maximal direction of stress in a circumferential direction and of strain in a perpendicular, longitudinal direction (Figure 3B–C). If we use elastic strain as a proxy for growth (see Discussion), the result of this simulation corresponds to the idea that organ growth is perpendicular to the fiber direction, so extending the organ along its main axis.

The experimentally observed circumferential direction of fibers seems to be explainable equally well by either the model where fibers orient perpendicularly to the direction of maximal strain (OsD) or by the model where fibers orient in the direction of maximal stress (MSD); both have been suggested as informative signals for fiber directions in plant tissues [3], [23], [24], [40]. To analyze the different consequences of these different variables acting as signaling cues for the fiber directions, we introduced a dynamic description of the wall material properties, where the deposition of new cellulose fibers leads to changes in magnitude and direction of the mechanical anisotropy of plant walls (Methods, Equations 8–10). We assumed a constant addition of fibers to the walls with the anisotropy of the deposition guided by the anisotropy of the directional signal, i.e if the input signal is isotropic, the material will be isotropic, while an anisotropic input signal will result in an anisotropic material.

Interestingly, the MSD and OsD hypotheses gave very different results, in spite of the fact that maximal strain and stress directions were perpendicular when fiber directions were fixed as descibed above. In the case of the stress based feedback, the fiber direction was identical to the fixed anisotropy direction case (Figure 3E–F), whereas in the case of (orthogonal) strain based feedback the initial, circumferential fiber direction became unstable and subsequently reorganized into the longitudinal direction (Figure 3H–K, Video S1), in contrast to the circumferential orientation of microtubules observed in experimental data. A more detailed analysis of the influence of material and loading force anisotropy on the MSD and OsD material models showed that the former model results in regions of mutually parallel and orthogonal strain and stress (Figure 3D). The extension of the region with perpendicular stress and strain directions was similar to the static anisotropy direction case (Figure 3A,D), indicating that orthogonal directions of maximal stress and strain constitutes a robust stable situation for the MSD dynamical model (Figure 3E, Figure S3). In the OsD model, the region of orthogonality between stress and strain disappeared completely (Figure 3G), indicating that this is an unstable situation for the OsD dynamical model (Figure S3). Independently of the anisotropy of the forces causing elastic deformation, the maximal stress and strain directions always became parallel (Figure 3H–K).

In conclusion, we have shown that in a situation where internal tissue is providing tension to the epidermis, an extension along the longitudinal axis of the organ can be explained by fibers resisting strain in the circumferential direction. This was clearly seen in a model where static fibers were laid out according to the experimentally observed pattern that results in a maximal strain that is orthogonal to the fiber direction. When the fiber directions were allowed to be reoriented by mechanical cues, more intricate dynamics was generated. A model where fibers aligned in the direction of maximal stress robustly preserved the circumferential directions of the fibers, as seen in experiments. On the contrary, a model where fibers aligned perpendicularly to the maximal strain direction led to the initial circumferential fiber pattern becoming unstable and reorienting into the longitudinal direction.

### A stress feedback model results in a radial zonation and can explain strain patterns in the shoot apical meristem

**Figure 4:**
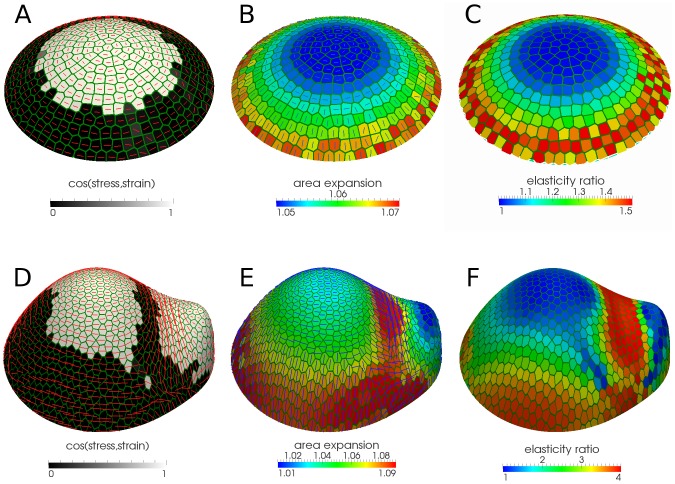
Zonation properties of the stress feedback model in meristem-like geometries. (A) The stress feedback together with fiber model for a paraboloid representing the geometry in the central zone and its close neighborhood results in two distinct zones where maximal stress and strain directions are either parallel (white) or perpendicular (black). The red bars(here and panel D) show fiber directions (B, C) Area expansion and material anisotropy (elasticity ratio) show different properties in these two regions. The elastic deformation is larger and radially oriented in the peripheral zone and the material is anisotropic whereas in the central zone deformation is less and the material becomes more isotropic. The blue lines (in the panels B and E) are showing the maximal strain directions. (D, E, F) The same results as A, B and C respectively for a meristem-like template. Maximal strain and stress directions are aligned at the apex and valley because of almost isotropic material and anisotropic stress respectively. For the meristem-like template due to the large variability of stress value in different regions the absolute stress anisotropy measure with 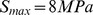 is used. The parameters used for pressurized templates in Figure 1E were: thickness 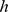 = 1 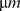, cell size about 10 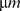, 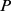 for paraboloid = 0.05 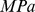 and for meristem = 0.08 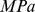, 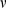 = 0.2, 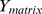 for paraboloid = 40 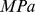 and for meristem = 50 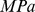, 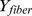 for paraboloid = 100 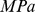 and for meristem = 150 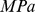, fiber model with 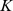 = 0.4, 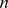 = 2. The deformation is within 5% to 7% for paraboloid and within 1% to 9% for meristem.

To test the dynamic stress feedback fiber model on a template with varying curvature, we applied the tissue pressure model to a paraboloid template, as a proxy for a naked meristem, in which the curvature is isotropic at the apex and smoothly becomes anisotropic across the periphery (Figure 1E). The dynamic changes of material properties in the cells resulted in a region of isotropic material at the apex and anisotropic material towards the periphery (Figure 4C), corresponding to isotropic stresses at the apex and anisotropic stresses in the periphery (Figure S5B). The dominant fiber direction oriented circumferentially around the central zone (Figure 4A), as previously reported in experiments and models [3], [40]. Remarkably, the switch from isotropic to anisotropic material (and stresses) in the radial direction was quite rapid, so creating a spontaneous zonation within the meristem purely from mechanical interactions. This corresponds to the very sharp transition between regions of parallel and perpendicular alignment of maximal stress and strain in the parameter space of material and loading force anisotropy (Figure 4A, D, cf. circles in Figure 3D). Therefore, even though these parameters change smoothly in the radial direction of the meristem, the dynamic material model creates an abrupt transition between the regions. The extent of these regions depended on model parameters, but the switch-like behavior was a robust feature of the stress feedback model (Figure S4).

The meristem has a central zone with slowly growing and dividing cells, and a peripheral zone where cells grow more quickly [41]–[43]. The cell expansion rates in the simulations also reflected the zonation (Figure 4B). The model predicted a slow isotropic expansion rate in the central zone and a comparatively high radially oriented expansion rate in the periphery, correlating well with strain directions reported for meristems [42].

Next, we looked in more detail on the effects of the dynamic update of material anisotropy direction and intensity on a geometry where there is a primordium at the periphery of the meristem with a valley in between (Figure 1E). Previously, we have shown that a tissue pressure model of the epidermis applied to a meristem shape leads to isotropic stress in the central zone while a valley in between the meristem and a primordium develops anisotropic stress. A simple spring model using a stress feedback generated fiber directions comparable to the measured microtubule directions in different areas of the meristem [3]. In the TRBS model, the stress feedback generated similar material fiber patterns (Figure 4D, Video S2), while the orthogonal strain feedback failed to generate these directions (Figure S5E, Video S2). In the valley between the meristem and the primordia, the stress feedback resulted in a fiber alignment along the valley and a high stress anisotropy (Figure S5D): the model predicted parallel maximal stress and strain directions in the valley (Figure 4D). While the alignment between maximal stress and strain directions in the central zone is a consequence of isotropic stresses in this region, alignment in the valley, in spite of anisotropic material, is caused by highly anisotropic stress (Figure 3D, blue and red circles, respectively), resulting in a maximal strain direction along the valley. Maximal directions of stress and strain were perpendicular elsewhere (Figure 4D, cf. Figure 3D, green and yellow circles), the same as in the periphery of the naked meristem simulation (Figure 4A).

In summary, we have shown that a stress feedback model is able to explain the patterns of microtubular organization seen in experiments. This feedback generates a relatively sharp zonation within the meristem, providing a purely mechanics-based explanation of strain magnitudes and directions inferred from experiments, where the central zone has a lower rate of isotropic expansion and the periphery a higher rate of radially directed expansion in spite of the circumferential stress direction. The model also predicts that highly anisotropic stresses generated in the boundary between the meristem and a primordium can lead to a maximal strain direction parallel to the maximal stress direction in this region.

### Stress and strain based feedback mechanisms have different impact on tissue geometry

Next we analyzed how dynamic properties of wall material affect elastic deformations locally and at a tissue scale. When anisotropic forces are applied (i.e. when curvature is higher in one direction in our tissue pressure models), the stress feedback model always aligns the fibers parallel to the maximal force so reducing the deformation in this direction and procuring locally a more isotropic deformation. Even in the case with strong anisotropy of the loading forces where maximal strain and stress are parallel (Figure 3Bi, cf. boundary region between meristem and primordia) the stress feedback model leads to a more isotropic strain field compared to an isotropic material of the same total elasticity. Since the strain feedback model aligns the fibers perpendicularly to the loading forces, this feedback tends to increase local strain anisotropies.

**Figure 5:**
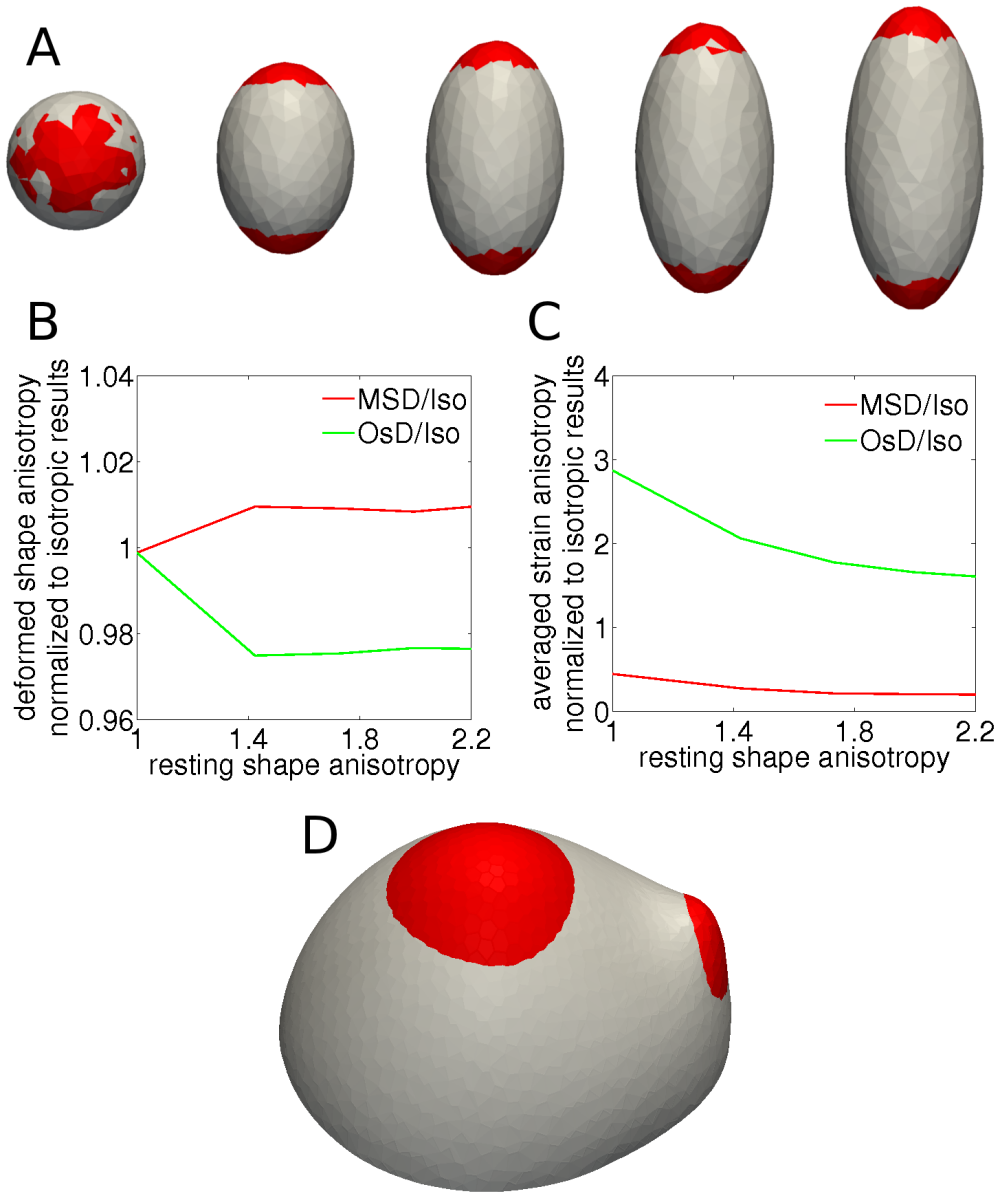
Stress and orthogonal strain feedback models impact on geometry. (A, B, C) Comparing stress and orthogonal strain feedback models for a set of templates with different geometric anisotropies which is considered here as the ratio between principal axes. This ratio is 1 for the sphere and increases for more elongated templates. (A) Higher anisotropic growth can be seen for the stress feedback model (red) compared to orthogonal strain feedback model (white). (B) The deformed shape anisotropy versus resting shape anisotropy for different feedback models. Values are normalized corresponding simulations with isotropic material of the same overall elasticity. The results show that even for a low deformation the stress feedback model increases shape anisotropy whereas orthogonal strain feedback model decreases this value, indicating that strain based feedback results in more symmetric geometry. (C) Strain anisotropy averaged over elements for simulations with the two feedback mechanisms are plotted versus resting shape anisotropy. The values are normalized to the corresponding simulations with an isotropic material of the same overal elasticity. In case of stress feedback the results are consistently lower than orthogonal strain feedback. (D) Comparing deformations resulting from different feedback models for the meristem-like pressurized template with the same parameters as Figure 4. More anisotropic growth in the stress feedback model (red) compared to the orthogonal strain feedback model (white) promotes the outgrowth of the primordium. The material parameters used in simulation were: 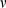 = 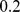, thickness 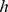 = 0.01 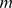, pressure 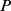 = 1.5 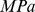. The radius of the sphere is 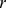 = 1 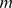, isotropic 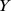 = 8 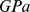, 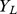 = 12 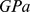, 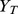 = 4 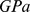.

To quantify these differences, we tested both material anisotropy feedback mechanisms within the TRBS model where geometries with different degrees of shape anisotropy were pressurized (Figure 5). When compared to isotropic material, the stress feedback model led to more isotropic strain, and the difference increased with the anisotropy of the geometry and hence with the loading force (Figure 5C). In contrast, the orthogonal strain based feedback model led to increased strain anisotropy when compared to an isotropic material (Figure 5C). This local difference had an impact on the resulting global deformation of the structure, where the stress feedback model promoted the maintenance of the geometrical anisotropy while the orthogonal strain feedback model decreased this anisotropy (Figure 5A–B).

Next we tested the different feedback models on our meristem-like template. The stress feedback model resulted in a more prominent anisotropic shape change at the meristem and primordium apices, promoting the upward movement of the shoot and a more directed shape change of the primordium (Figure 5D). Also, the stress based feedback model resulted in a more pronounced valley between the meristem and the new organ (Figure 5D, Video S2). The changes of these features of the meristem have been seen experimentally when comparing wild-type plants and plants treated with oryzalin, a drug that depolymerizes microtubules and is assumed to lead to a more isotropic material [44].

In summary, a stress feedback to fiber directions enables plant walls to resist internal forces, which locally generates more isotropic elastic strains, and which at the same time counteracts to the tissue pressure forces acting towards isotropic curvature and hence a stress feedback maintains the shape of anisotropic structures.

## Discussion

The coordination of the changes in mechanical properties across a growing plant tissue is crucial for the creation of the complicated forms and shapes observed in plants [7], [21]. In the shoot apical meristem a connection between the organization of the tissue's mechanical anisotropy and the perception of mechanical stress signals has been suggested [3], [40], while a competing idea that fibers organize perpendicular to the strain direction has emerged motivated by the correlation between growth and fiber directions in anisotropically growing organs [23], [24]. In this study we analyzed two models in order to compare these mechanisms for how mechanical cues feed back to orient cellulose microfibers. In our simulations of the stress and strain patterns in the epidermis on a stem-like geometry, where fibers are fixed to be aligned circumferentially, maximal stress is circumferential and maximal strain is longitudinal, in accord with both experimentally motivated suggestions for organizing fiber directions. However, in models of dynamic orientation of cell wall mechanical anisotropy driven by stress or strain, we observed drastically different results for each of the two models. In the stress based feedback model the circumferential alignment of the fibers as well as the perpendicular orientation of maximal stress and strain directions can be robustly maintained (Figure 3E). In contrast, the orthogonal to strain based feedback model results in a longitudinal alignment of the fibers and parallel, circumferential directions of maximal strain and stress, which contradicts the experimentally observed orientation of the cellulose fibers and microtubules (Figure 3H).

When simulating the more complex shapes appearing around the shoot apical meristem, the orthogonal strain feedback model again failed to explain the microtubule patterns seen in experiments. The stress feedback model translated the smooth increase of anisotropic curvature in the radial direction to a switching between different material properties in a central and a peripheral zone (Figure 4). It should be noted that even if there is an instant shift of strain and stress directions from mutually parallel to perpendicular, this does not represent a discontinuity in the model since the strain direction is degenerate (isotropic) when crossing these boundaries (Figure S5A,C). A mechanical radial zonation has recently been suggested by experiments and models [32], [45], but in our model different properties of the material in different areas of the tissue are not dictated by an arbitrary specification of the separate regions. They are instead a natural consequence of the stress feedback model reacting to the differences in shape, curvature and stress response and stress anisotropy in different regions of the meristem. The alignment of maximal stress and strain directions in the central zone is a consequence of the material being isotropic in this region. The analogous alignment in the valley between the shoot and a primordium, which occurs in spite of the material's anisotropy, is caused by a highly anisotropic stress. The perpendicular maximal stress and strain directions in the periphery are a result of an anisotropic material and anisotropic forces, but where the forces are sufficiently opposed by the fibers to create a perpendicular strain direction. Such radial growth direction has been reported in experiments [42].

It is interesting to relate the spontaneously formed mechanical patterns to the known radial expression patterns in genes regulating differentiation [46]. It has been recently shown that the stem cell regulator WUSCHEL, expressed in the central regions of the shoot, moves between cells and directly activate the stem cell marker CLAVATA3, expressed in the apical region of the meristem. WUSCHEL also represses genes that are important for differentiation. The combined gene regulatory network is sufficient to explain the radial expression zonation in the meristem [47]. How this molecular network interacts with mechanical properties is an interesting question for the future. While there might not be a direct interaction, both the mechanical and molecular models do depend on the shape of the meristem to generate a radial zonation and can hence affect each other's radial zonation via the geometry changes. Our simulations performed on templates resembling the shapes of the stem and meristem with outgrowing primordia show that a stress based feedback produces deformations which result in more elongated shapes of outgrowing organs while an orthogonal to strain feedback tends to round and level the protrusions of the surface (Figure 5, Video S2). Interestingly, this is a consequence of the stress based feedback having more isotropic strain locally, compared to an isotropic material or a strain based feedback mechanism.

We have compared the results of continuum mechanics simulations using a Triangular Biquadratic Spring method with more detailed simulations using a nonlinear shell Finite Element method. We found that the methods are in agreement for both stretching and bending pressurized tissue simulations that are used to represent epidermal plant tissue. This shows that TRBS, despite its simplified treatment of geometry and its lack of bending resistance, offers an adequate level of accuracy for the purpose of modeling plant tissue. Owing to its simplicity the TRBS method will prove useful for more complicated three dimensional models involving cell growth and proliferation and thus requiring changes in model topology.

The assumption of modeling the internal cell layers as a simplified tissue pressure contribution can be analyzed further in future work, which can allow for a more complex interaction between internal layers and the epidermis in the meristem [9], [11]. Our simulations suggest that this will improve the description mainly in situations with a negative curvature and compressive forces, e.g. in the boundary between the meristem and a primordium. Our simulations overestimate the strain rates in these regions and the absence of internal tissue can lead to buckling (Figure S2E). For anisotropically shaped organs, this may also be important for the stress directions. For stem tissue it has been shown that the internal tissue exerts a longitudinal force on the epidermis with longitudinally oriented fibers [48]. The stress feedback model applied to our cylindrical template in the presence of large logitudinal forces will lead to fibers in the longitudinal direction (Figure S6, cf. red circle in Figure 3D), which is in accord with patterns seen in experiments [49]. However, the appearance of anisotropic longitudinal growth in internal tissues is still not understood in such a scenario [48], and will probably require more data on fiber orientations in several cell layers [38].

Another challenge will be to integrate current models with long-term plastic growth of plant cell walls. Plastic growth is described as being triggered by wall stresses above a yield stress, which induces a break and slip behavior [50], while we have compared elastic strain in the simulations with the plastic growth in experiments. While this might seem to be a contradiction, as we show that often the maximal stress and strain directions are perpendicular, it would be easy to remedy this difference. Either stresses in the isotropic matrix part of the wall could be used, which is the same as the strain, or the growth direction could follow the maximal stresses, but be oriented perpendicular to the fibers. Interestingly, our model predicts that a matrix stress idea and a stress perpendicular to fibers idea for growth can be discerned by a detailed measuring of growth directions in the boundary between the meristem and the new primordia, since there the fiber and strain directions are parallel. In any scenario, the maximal stress direction would not provide a good cue for plastic growth, since this would counteract the possibility to generate anisotropically shaped organs.

There are no experimental molecular data on how stress or strain sensing mechanisms work, although several suggestions have been proposed for how it could be realized [7], [51]. The recent data show that microtubule-severing protein katanin is required for the cell's response to mechanical signals in plants [26], and several examples in animals show that proteins can act as mechanosensors, e.g. [52].

The development of detailed mechanical models will be integral for understanding morphogenesis in development. It will open up new venues of research for understanding whether mechanical cues are one of the main drivers of shape changes, and more importantly it will allow the development of integrated models where gene regulation and molecular signaling feed back to each other for describing the combined effects of differentiation and morphogenesis.

## Models

### Material models of anisotropic tissue

There exist many material models which parametrize elastic energy in terms of combination of deformation tensor invariants in different ways and describe behavior of different types of materials. In the simplest isotropic material case the TRBS uses a St. Venant-Kirchoff description, which is an extension of a linear material model. The strain energy density, 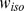, in this material model becomes
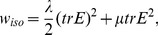
(1)where 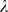 and 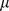 are Lame coefficients representing material elasticity and 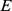 is the Green-Lagrange strain tensor. The advantages of this material model are the simple energy form and a clear interpretation of material properties. We assume plane stress condition where Lame constants can be expressed as
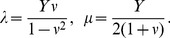
(2)Here 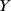 and 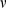 are the Young modulus and Poisson ratio that represent elasticity and incompressibility of the material, respectively.

In order to extend this material model for transversely isotropic materials we considered two sets of Lame constants, one for longitudinal and one for transverse to anisotropy direction [53]. To ensure that the energy expression is not over-penalized in the anisotropy direction we first equipartitioned the energy into three terms each corresponding to one of the principal directions (longitudinal and two transverse directions with respect to fibers). Then we have penalized only the term corresponding to the anisotropy direction. A procedure which do not take into account equipartitioning of the energy overestimates the contribution of the anisotropic part [53]. The increased energy cost of deformation in direction of the fiber, 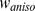, is then described by
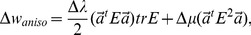
(3)where the anisotropic part contains invariants of a strain tensor 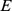 constructed with a vector in the direction of the fibers 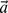. The 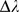 and 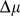 are the differences between longitudinal and transverse Lame coefficients which are in turn related to Young modulus in longitudinal and transverse directions (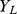, 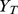) and Poisson ratio
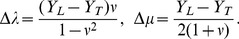
(4)where 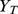 follows similar relation as 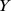 in Equation 2 and is representing the elasticity of the material in the transverse directions. The total energy density, 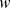, including an isotropic term for the matrix and an anisotropic term for the fiber becomes
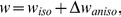
(5)which can be used for calculating the stress tensor and forces applied on the nodes of the meshed structure (Text S1).

### Evaluating strain and stress and their anisotropy

The expression for St. Venant-Kirchoff energy (Eq. 1) is based on the Green-Lagrange strain tensor, 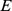, which can be expressed in terms of a deformation tensor. The second Piola-Kirchhoff stress tensor, which is the energy conjugate of the Green-Lagrange strain tensor, yields the stress in the resting shape. For evaluating strain and stress in the deformed shape, which is the current configuration, we calculated Almansi strain and its energy conjugate, Cauchy or true stress tensors, respectively (Text S1). The stress in case of TRBS was calculated under the assumption of plane stress and in case of shell description we visualized the stress integrated over thickness in order to be comparable to the corresponding values for TRBS.

All of these tensors are two dimensional for TRBS elements and three dimensional for shells. The relative stress (strain) anisotropy measure, 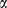, can be defined as
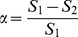
(6)where 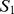 and 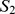 are first and second stress (strain) eigenvalues respectively. We consider only tensile stress (strain) to be relevant and compressive stress (strain) values were set to zero in the equations.

In most simulations the magnitudes of stresses (strains) are of the same order of magnitude and such relative measure is appropriate. However, in the case of our meristem-like template with outgrowing primorium, stress (strain) magnitudes extend over a large range. In such a scenario, a relative value can overestimate an anisotropy measure in regions of low stresses (strains), and it is more appropriate to include the stress (strain) magnitude itself in the measure. In this case we used
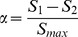
(7)where 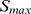 is the largest stress (strain) value throughout the template (excluding boundary effects). We have normalized the value of anisotropy measure since we use as an input to the material model an expression assuming the value of this parameter to be between 0 and 1 (see next section). In most cases the anisotropy measures based on both definitions follow the same trend but in more complicated geometries, where strain and stress values are small, there can be significant differences between the two measures (e.g. the primordial apical region in the meristem-like template).

### Fiber model and updating material properties

**Figure 6:**
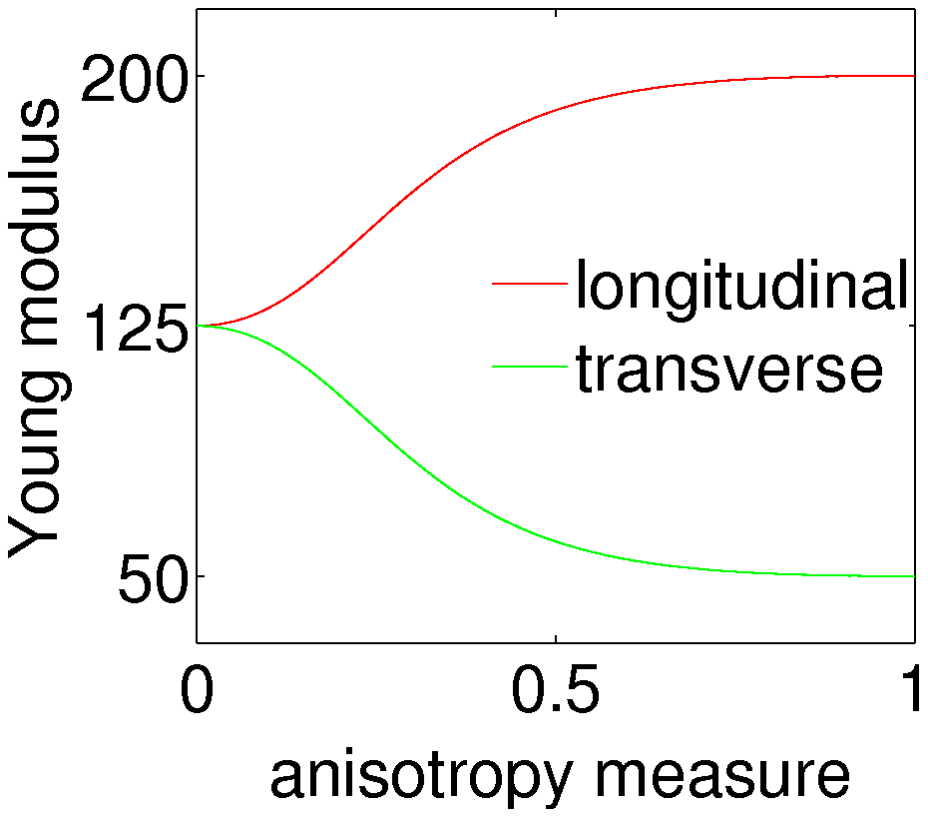
The fiber model. In the fiber model mechanical anisotropy is adjusted based on an anisotropy measure dependent on stress or strain in such way that the overall elasticity of the material is conserved. The plot shows result of using 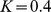, 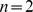 and 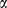 between 0 and 1 in Equation 7. In our simulations model parameters were chosen such that material anisotropy was close to its maximum when stress anisotropy was about 0.5.

Since plant tissue is characterized by different and dynamically changing anisotropic material properties we have devised a model which allows for smooth temporal and spatial changes of anisotropy. The model assumes that stress anisotropy plays a role in defining the degree of material anisotropy while the average elastic strength of the material is maintained. We used a non-linear relation between stress and material anisotropy which saturates when stress anisotropy is maximal (Figure 6). The relations between longitudinal, 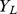, and transverse, 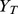, Young modulus and anisotropy measure, 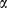, can be written as
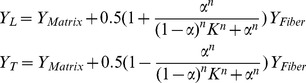
(8)where 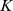 and 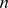 are model parameters and 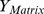 and 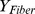 are Young moduli of the isotropic matrix and anisotropic fiber part respectively.

We implemented a delay in the update of longitudinal and transverse Young moduli (fiber model) as well as anisotropy direction of individual cells (stress feedback) to take into account different time scales of propagation of mechanical and biochemical interactions. Such approach also results in more stable simulations. The Euler steps for updating longitudinal and transverse Young modulus are
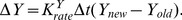
(9)where 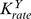 determines the time delay, 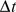 is the time step, 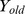 is the current value and 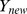 is the new values calculated from Equation 8. Similarly the update for anisotropy direction is done based on
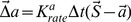
(10)where 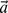 is the current anisotropy direction vector and 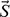 is the maximal stress direction vector, and 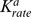 again sets the time delay.

### Mechanical simulations

The mechanical simulations have been performed with in house developed software optimized for simulations of cellular structures. Both methods used in our simulations (TRBS and shells) are based on the FEM approach, which relies on the division of the domain of interest into simpler geometrical elements (meshing) and looking for the solution of the continuous mechanics equations in the basis of the functions which are local to each element. In case of shell FEM simulations we have used quadrilateral shell elements within extensible director formulation [28] (Figure 1A). The implementation of TRBS was based on the explicit procedure used previously in simulation of biological materials [30] (Figure 1B). We triangulated the polygonal cells via adding a vertex at the centroid position. Since we used a single fiber direction in cells, we averaged stress or strain input from the individual triangles. In our simulations both explicit Newark and implicit solvers with Newton-Rapson iteration were used for the shell finite element implementation while explicit forth order and adaptive fifth order RungeKutta methods were used for TRBS. The material parameters used in the simulations of plant-like structures (Figures 3 and 4) were matched to the experimental estimates from similar materials [34]–[36]. We have used Young modulus in range 40 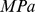
–50 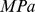 and 100 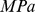
–120 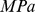 for isotropic and anisotropic part of the material, respectively. Poisson ratio was set to 0.2 and turgor pressure 0.2 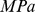. We assumed a thickness of epidermal material of 1 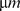 and a cell size of order 10 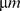 to 20 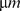. In the fiber model we have used 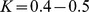 and 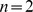. For updating anisotropy directions and material properties using the equations 8 and 9 we used 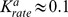 for anisotropy direction update and 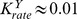 for material properties update. As long as small values for update rates were used the results were not sensitive to the exact value of those parameters. We have used smaller update rates for material properties update, assuming the change in material properties is a consequence of microtubular dynamics and should be delayed respect to the anisotropy direction update. These parameters resulted in the deformation of order 5% to 10% in agreement with experimentally reported estimates [32]. We have used fixed (clamped) boundary conditions for our simulations of pressurized templates, which means that there was no deformation on the open boundary edges of the simulated structures. Since such conditions are not exact for real plant organs and can affect the results of simulations close to the boundary we excluded those regions from the analysis. The effects of the boundary conditions can be seen in Video S2.

### Availability

The simulation tools are in house implementations and the latest versions are publicly available via a subversion server upon request and the current versions are available as Supplemental Information.

Operating system(s): Platform independent;
Programming language: C++;
Licence: no licence needed.

## Supporting Information

Figure S1**Comparing triangular biquadratic springs and finite element shell models.** (A) The difference between principal stress value of triangular biquadratic springs model and integrated principal stress over thickness in finite element shell model is subtle ( less than 0.1%) ‘Shell’ elements for the case of isotropically loaded patch (compare with Fig. 2C, model parameters are the same in two models). (B) The first and second principal stress values for our anisotropic material model become more different as material becomes more anisotropic.The patch was isotropically loaded constantly with 2 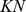 when material anisotropy was varied by keeping the transverse Young modulus constant (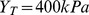) and changing the fiber Young modulus (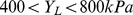) for Poisson ratio(P = 0.3). Elasticity ratio is the ratio between longitudinal and transverse Young moduli. (C–E) Comparison between principal stress direction and principal strain value in two models(left: TRBS, right: shell) for different pressurized templates show a major similarity indicating the lack of bending energy in TRBS model is not important when deformation is caused by internal pressure. (C) Isotropic material with Young modulus = 40 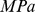, Poisson ratio = 0.2,Pressure = 0.01 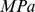 (D) Isotropic material with Young modulus = 90 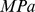, Poisson ratio = 0.2, Pressure = 0.1 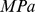 (E) Isotropic material with Young modulus = 80 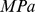, Poisson ratio = 0.2,Pressure = 0.05 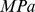 (F) for a saddle-like template where the compressive forces become important resulting in buckling, the difference in deformation in two models is obvious.Isotropic material with Young modulus = 40 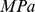, Poisson ratio = 0.2,Pressure = 0.01 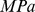.(PDF)

Figure S2**Poisson ratio analysis.** Similar plot as Figure 3A for different values for Poisson ratio (A) 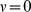, (B) 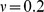, (C) 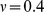. In the force/material anisotropy space the region where principal directions of stress and strain are perpendicular is larger for lower values of Poisson ratio.(PDF)

Figure S3**Fixed points of dynamical stress/strain based feedback updates of anisotropy direction.** For the quadrilateral patch of anisotropic material with constant transverse and longitudinal Young modulus as 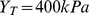 and 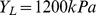 respectively and Poisson coefficient = 0.2 under anisotropic loading 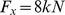 and 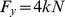 the direction of principal stress and perpendicular to the direction of principal strain are plotted versus the angle of varying anisotropy direction. 0 value for the angles is corresponding with maximal force direction. There is a fixed point at zero for both feedback systems which is stable for stress feedback whereas extremely unstable for the perpendicular to maximal strain feedback.(PDF)

Figure S4**Different zonation resulting from fiber model with different values for the K parameter.** (A) 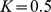, (B) 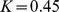, (C) 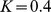.(PDF)

Figure S5**Additional meristem-like template simulations.** (A–D) Additional information about Figure 4. (E) Anisotropy direction pattern for the same simulation as Figure 4D–F, using perpendicular to strain feedback model and the fiber model with strain anisotropy measure. All of the model parameters are the same except strain constant in Equation 6, 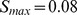.(PDF)

Figure S6 **Effect of axial loading.** Adding regional axial tensile stress (red arrows) to the Tissue Pressure model in different feedback scenarios. Axial stress is applied so that in the region between red arrows maximal stress is axial with stress anisotropy about 0.6–0.7. In other regions maximal stress is circumferential with stress anisotropy about 0.5. (A) Stress feedback. (B) Perpendicular to strain feedback. Material properties and pressure are the same as Figure 3.
(PDF)

Software S1 **Archive file (tar.gz) including source code for the in-house developed C++ simulation software used in this study.** Also provided are instructions and scripts for generating all results of the paper. Follow instructions in the README.txt file in the top directory for how to compile the software and generate the results.
(GZ) Click here for additional data file.

Text S1 **Supporting information.** Additional information about continuum mechanics methods used in the paper and extended model description.
(PDF)

Video S1 **Difference in dynamical deformation and changes in anisotropy directions of cells in different feedback models using the cylindrical template with initial circumferential orientation of anisotropy directions with the same model parameters as ****Figure 3****.** Left: stress feedback. Right: perpendicular to strain feedback. Stress feedback results maintain the circumferential pattern and consequently more elongated deformation whereas the feedback model based on perpendicular direction to maximal strain rapidly breaks the pattern and rearranges them along the cylinder causing increase of the width of the cylinder.
(MOV)

Video S2 **Difference in dynamical deformation and changes in anisotropy directions of cells in different feedback models using the meristem-like template.** Left: stress feedback. Right: perpendicular to strain feedback. Stress feedback results in circumferential pattern and consequently more anisotropic deformation whereas the feedback model based on perpendicular direction to maximal strain causes a radial pattern for anisotropy directions and deforms the template towards more isotropic shape. Here the model parameters are modified to achieve larger deformations (up to 20%).
(MOV)
