# Supplementary material for: Stress and Strain Provide Positional and Directional Cues in Development
Source: PLoS Comput Biol. 2014 Jan 9;10(1):e1003410. doi: 10.1371/journal.pcbi.1003410 (PMC3886884; doi:10.1371/journal.pcbi.1003410)
Supplement: Figure S1 — Comparing triangular biquadratic springs and finite element shell models. (A) The difference between principal stress value of triangular biquadratic springs model and integrated principal stress over thickness in finite element shell model is subtle ( less than 0.1%) ‘Shell’ elements for the case of isotropically loaded patch (compare with Fig. 2C, model parameters are the same in two models). (B) The first and second principal stress values for our anisotropic material model become more different as material becomes more anisotropic.The patch was isotropically loaded constantly with 2 when material anisotropy was varied by keeping the transverse Young modulus constant () and changing the fiber Young modulus () for Poisson ratio(P = 0.3). Elasticity ratio is the ratio between longitudinal and transverse Young moduli. (C–E) Comparison between principal stress direction and principal strain value in two models(left: TRBS, right: shell) for different pressurized templates show a major similarity indicating the lack of bending energy in TRBS model is not important when deformation is caused by internal pressure. (C) Isotropic material with Young modulus = 40 , Poisson ratio = 0.2,Pressure = 0.01 (D) Isotropic material with Young modulus = 90 , Poisson ratio = 0.2, Pressure = 0.1 (E) Isotropic material with Young modulus = 80 , Poisson ratio = 0.2,Pressure = 0.05 (F) for a saddle-like template where the compressive forces become important resulting in buckling, the difference in deformation in two models is obvious.Isotropic material with Young modulus = 40 , Poisson ratio = 0.2,Pressure = 0.01 . (PDF) [file pcbi.1003410.s001.pdf]

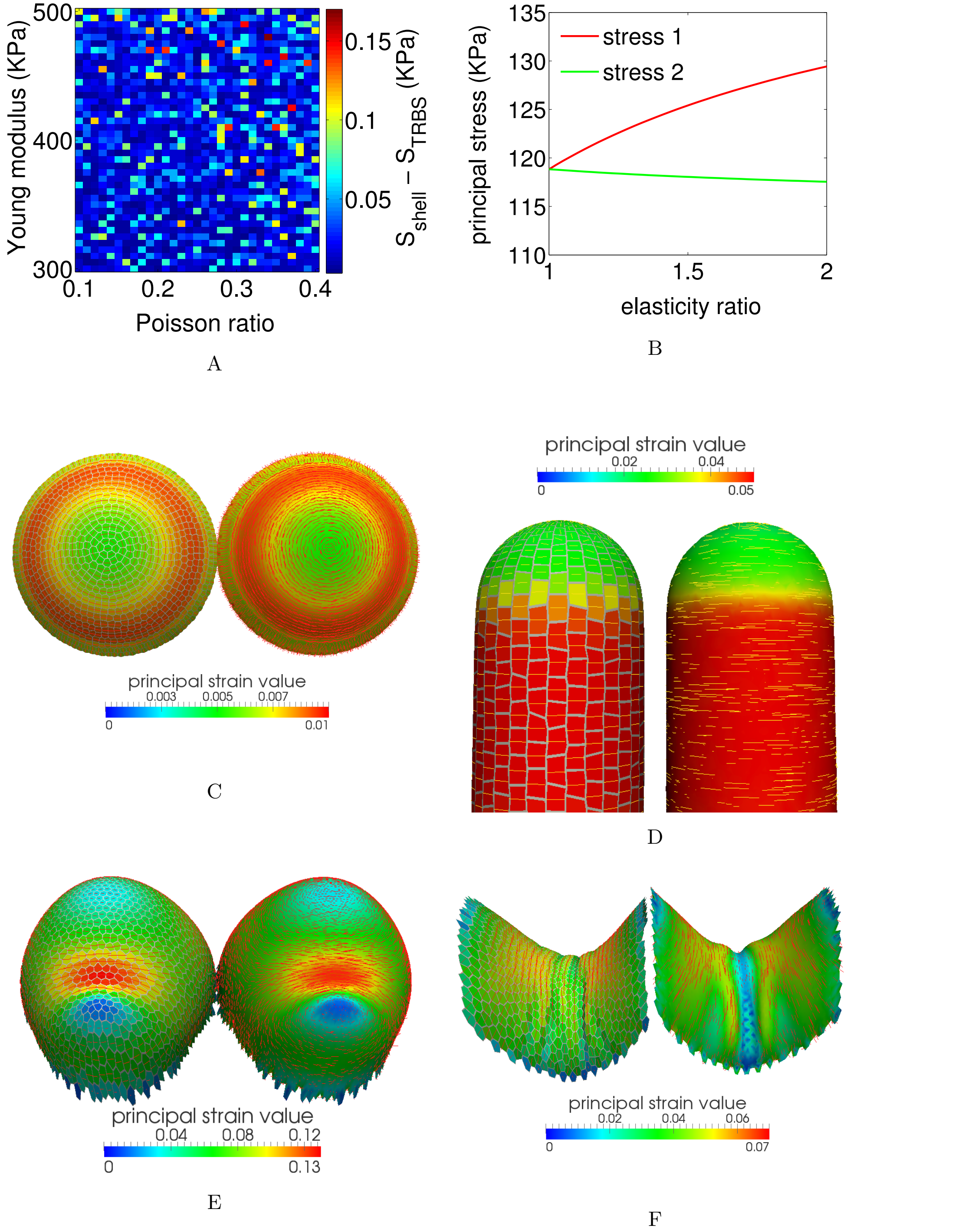

Figure S1: **Comparing triangular biquadratic springs and finite element shell models.** (A) The difference between principal stress value of triangular biquadratic springs model and integrated principal stress over thickness in finite element shell model is subtle (less than 0.1 %) 'Shell' elements for the case of isotropically loaded patch (compare with Fig. 2C, model parameters are the same in two models). (B) The first and second principal stress values for our anisotropic material model become more different as material becomes more anisotropic.The patch was isotropically loaded constantly with 2 KN when material anisotropy was varied by keeping the transverse Young modulus constant ($Y_T = 400kPa$) and changing the fiber Young modulus ($400 < Y_L < 800kPa$) for Poisson ratio(P=0.3). Elasticity ratio is the ratio between longitudinal and transverse Young moduli. (C-E) Comparison between principal stress direction and principal strain value in two models(left: TRBS, right: shell) for different pressurized templates show a major similarity indicating the lack of bending energy in TRBS model is not important when deformation is caused by internal pressure. (C) Isotropic material with Young modulus = 40 MPa, Poisson ratio = 0.2 ,Pressure = 0.01 MPa (D) Isotropic material with Young modulus = 90 MPa, Poisson ratio = 0.2, Pressure = 0.1 MPa (E) Isotropic material with Young modulus = 80 MPa, Poisson ratio = 0.2 ,Pressure = 0.05 MPa(F)for a saddle-like template where the compressive forces become important resulting in buckling, the difference in deformation in two models is obvious.Isotropic material with Young modulus = 40 MPa, Poisson ratio = 0.2 ,Pressure = 0.01 MPa
