# Supplementary material for: Stress and Strain Provide Positional and Directional Cues in Development
Source: PLoS Comput Biol. 2014 Jan 9;10(1):e1003410. doi: 10.1371/journal.pcbi.1003410 (PMC3886884; doi:10.1371/journal.pcbi.1003410)
Supplement: Figure S2 — Poisson ratio analysis. Similar plot as Figure 3A for different values for Poisson ratio (A) , (B) , (C) . In the force/material anisotropy space the region where principal directions of stress and strain are perpendicular is larger for lower values of Poisson ratio. (PDF) [file pcbi.1003410.s002.pdf]

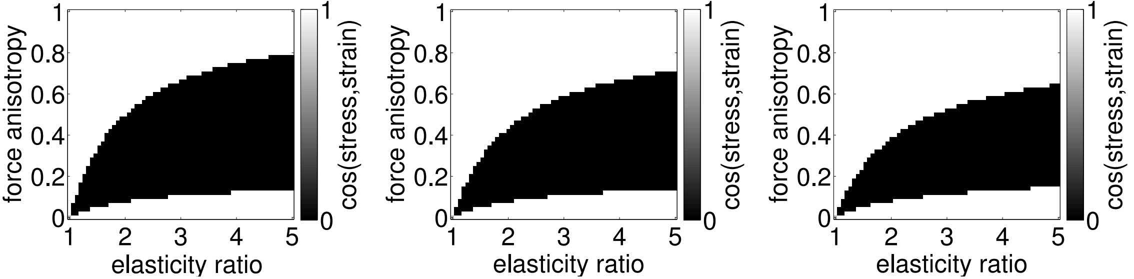

A B C

Figure S2: **Poisson ratio analysis** Similar plot as Figure 3A for different values for Poisson ratio (A) $\nu = 0$,(B) $\nu = 0.2$, (C) $\nu = 0.4$. In the force/material anisotropy space the region where principal directions of stress and strain are perpendicular is larger for lower values of Poisson ratio
