# Supplementary material for: Stress and Strain Provide Positional and Directional Cues in Development
Source: PLoS Comput Biol. 2014 Jan 9;10(1):e1003410. doi: 10.1371/journal.pcbi.1003410 (PMC3886884; doi:10.1371/journal.pcbi.1003410)
Supplement: Figure S3 — Fixed points of dynamical stress/strain based feedback updates of anisotropy direction. For the quadrilateral patch of anisotropic material with constant transverse and longitudinal Young modulus as and respectively and Poisson coefficient = 0.2 under anisotropic loading and the direction of principal stress and perpendicular to the direction of principal strain are plotted versus the angle of varying anisotropy direction. 0 value for the angles is corresponding with maximal force direction. There is a fixed point at zero for both feedback systems which is stable for stress feedback whereas extremely unstable for the perpendicular to maximal strain feedback. (PDF) [file pcbi.1003410.s003.pdf]

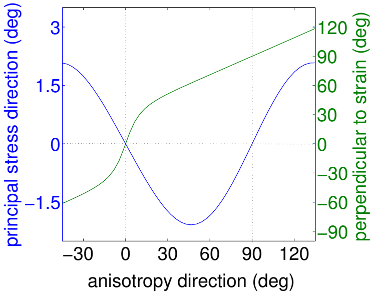

Figure S3: **Fixed points of dynamical stress/strain based feedback updates of anisotropy direction.** For the quadrilateral patch of anisotropic material with constant transverse and longitudinal Young modulus as $Y_T = 400kPa$ and $Y_L = 1200kPa$ respectively and Poisson coefficient = 0.2 under anisotropic loading $F_x = 8kN$ and $F_y = 4kN$ the direction of principal stress and perpendicular to the direction of principal strain are plotted versus the angle of varying anisotropy direction. 0 value for the angles is corresponding with maximal force direction. There is a fixed point at zero for both feedback systems which is stable for stress feedback whereas extremely unstable for the perpendicular to maximal strain feedback.
