# Supplementary material for: Stress and Strain Provide Positional and Directional Cues in Development
Source: PLoS Comput Biol. 2014 Jan 9;10(1):e1003410. doi: 10.1371/journal.pcbi.1003410 (PMC3886884; doi:10.1371/journal.pcbi.1003410)
Supplement: Figure S4 — Different zonation resulting from fiber model with different values for the K parameter. (A) , (B) , (C) . (PDF) [file pcbi.1003410.s004.pdf]

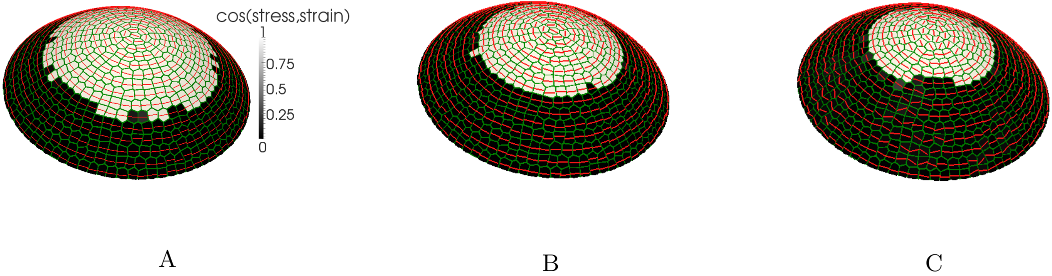

Figure S4: **Different zonation resulting from fiber model with different values for the K parameter.** (A) $K = 0.5$, (B) $K = 0.45$, (C) $K = 0.4$.
