# Supplementary material for: Stress and Strain Provide Positional and Directional Cues in Development
Source: PLoS Comput Biol. 2014 Jan 9;10(1):e1003410. doi: 10.1371/journal.pcbi.1003410 (PMC3886884; doi:10.1371/journal.pcbi.1003410)
Supplement: Figure S5 — Additional meristem-like template simulations. (A–D) Additional information about Figure 4. (E) Anisotropy direction pattern for the same simulation as Figure 4D–F, using perpendicular to strain feedback model and the fiber model with strain anisotropy measure. All of the model parameters are the same except strain constant in Equation 6, . (PDF) [file pcbi.1003410.s005.pdf]

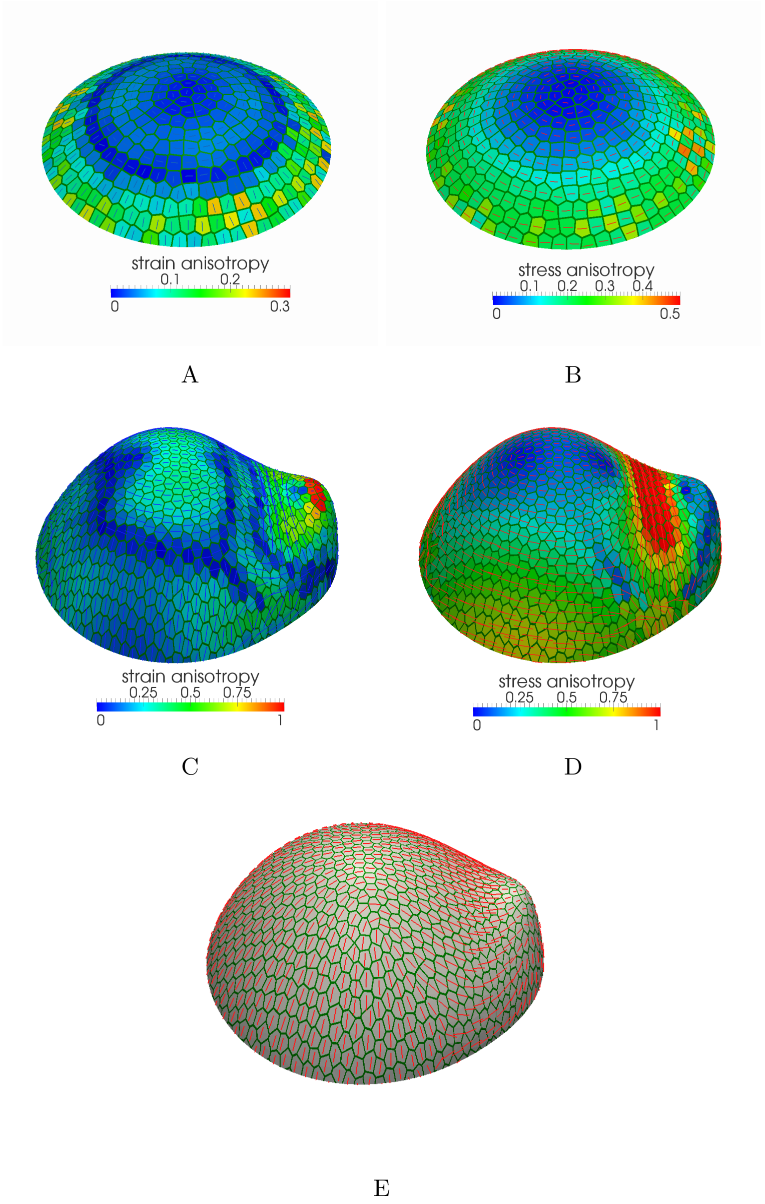

Figure S5: **Additional meristem-like template simulations.** (A-D) Additional information about Figure 4. (E) Anisotropy direction pattern for the same simulation as Figure 4D-F, using perpendicular to strain feedback model and the fiber model with strain anisotropy measure. All of the model parameters are the same except strain constant in Equation 6, $S_{max} = 0.08$
