# Supplementary material for: Stress and Strain Provide Positional and Directional Cues in Development
Source: PLoS Comput Biol. 2014 Jan 9;10(1):e1003410. doi: 10.1371/journal.pcbi.1003410 (PMC3886884; doi:10.1371/journal.pcbi.1003410)
Supplement: Figure S6 — Effect of axial loading. Adding regional axial tensile stress (red arrows) to the Tissue Pressure model in different feedback scenarios. Axial stress is applied so that in the region between red arrows maximal stress is axial with stress anisotropy about 0.6–0.7. In other regions maximal stress is circumferential with stress anisotropy about 0.5. (A) Stress feedback. (B) Perpendicular to strain feedback. Material properties and pressure are the same as Figure 3. (PDF) [file pcbi.1003410.s006.pdf]

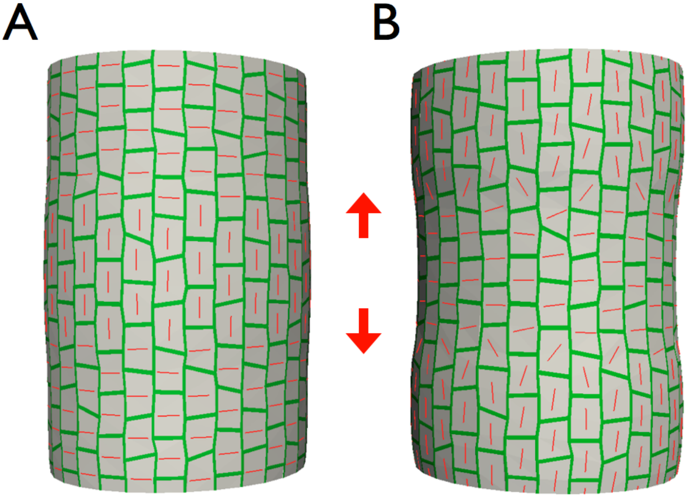

Figure S6: **Effect of axial loading.** Adding regional axial tensile stress (red arrows) to the Tissue Pressure model in different feedback scenarios. Axial stress is applied so that in the region between red arrows maximal stress is axial with stress anisotropy about 0.6-0.7. In other regions maximal stress is circumferential with stress anisotropy about 0.5. (A) Stress feedback. (B) Perpendicular to strain feedback. Material properties and pressure are the same as Figure 3.
