# Supplementary material for: Stress and Strain Provide Positional and Directional Cues in Development
Source: PLoS Comput Biol. 2014 Jan 9;10(1):e1003410. doi: 10.1371/journal.pcbi.1003410 (PMC3886884; doi:10.1371/journal.pcbi.1003410)
Supplement: Text S1 — Supporting information. Additional information about continuum mechanics methods used in the paper and extended model description. (PDF) [file pcbi.1003410.s008.pdf]

Supporting information

1 Strain energy formalism

1.1 Isotropic strain energy

The model that we used, as most continuous mechanics methods, is based on the minimization of strain energy. In the case of St. Venant-Kirchoff description for the isotropic material [1], this energy takes the form

$$W_{iso} = \int_{\Omega} w_{iso} d\Omega = \int_{\Omega} (\frac{\lambda}{2}(trE)^2 + \mu trE^2) d\Omega, \quad (1)$$

where Ω is the material domain, E is a Green-Lagrange strain tensor and Lame parameters λ and μ are related to Young's modulus Y and Poisson's ratio ν. In three dimensions the relation between those material parameters becomes [1]

$$\lambda = \frac{Y\nu}{(1+\nu)(1-2\nu)}, \quad \mu = \frac{Y}{2(1+\nu)}. \quad (2)$$

In case of plane stress condition these equations take form [1]

$$\lambda = \frac{Y\nu}{1-\nu^2}, \quad \mu = \frac{Y}{2(1+\nu)}. \quad (3)$$

The Green-Lagrange strain tensor can be expressed in terms of Cauchy-Green deformation tensor C and identity tensor I as

$$E = \frac{1}{2}(C - I). \quad (4)$$

The Cauchy-Green deformation tensor is a function of deformation gradient tensor F

$$C = F^T F. \quad (5)$$

The deformation gradient tensor F is the gradient of the deformation function Φ

$$F = \nabla \Phi \quad (6)$$

If we describe a motion of the particle as

$$\mathbf{x} = \mathbf{\Phi}(\mathbf{X}, t), \tag{7}$$

where $\mathbf{x}$ is a position vector of particle in current configuration and $\mathbf{X}$ its position in undeformed (material) coordinates.

The strain energy density is usually expressed in terms of invariants of the strain tensor $I_1 = trE$ and $I_2 = trE^2$

$$w_{iso} = \frac{\lambda}{2} I_1^2 + \mu I_2, \tag{8}$$

or equivalently by Cauchy-Green tensor invariants $I_1' = trC$ and $I_2' = trC^2$

$$I_1 = \frac{1}{2} I_1' - \frac{1}{2} trI, \tag{9}$$

$$I_2 = \frac{1}{4} I_2' - \frac{1}{2} I_1' + \frac{1}{4} trI. \tag{10}$$

1.2 Anisotropic strain energy

Strain energy density for the isotropic material can be decomposed as:

$$\begin{aligned} w_{iso} &= w_x + w_y + w_z \\ &= \frac{\lambda}{2}(e_1^T E e_1) trE + \mu(e_1^T E^2 e_1) \\ &+ \frac{\lambda}{2}(e_2^T E e_2) trE + \mu(e_2^T E^2 e_2) \\ &+ \frac{\lambda}{2}(e_3^T E e_3) trE + \mu(e_3^T E^2 e_3), \end{aligned} \tag{11}$$

where the e_i, for $i = 1...3$ are versors of Cartesian coordinate system and three terms present the equal parts corresponding to each of the x, y and z directions. Analogically the energy w_a corresponding with arbitrary direction represented by the vector $\vec{a}$ we expressed as

$$w_a = \frac{\lambda}{2}(\vec{a}^T E \vec{a}) trE + \mu(\vec{a}^T E^2 \vec{a}). \tag{12}$$

Now if we consider a transversely isotropic material, which has different mechanical properties in a single direction we can define

$$\Delta\lambda = \lambda^L - \lambda^T \tag{13}$$

and

$$\Delta\mu = \mu^L - \mu^T, \tag{14}$$

where λ^L, μ^L are *Longitudinal* Lame constants in a given direction $\vec{a}$ and λ^T, μ^T are *Transverse* Lame constants in a plane transverse to $\vec{a}$. These can be related to the longitudinal Y_L and transverse Y_T Young modulus by the use of Equation 2 or 3 and considering the same Poisson's ratio for different directions (Equation 4 in the main text shows this for plane stress).

With these definitions we introduced the term which has to be added to isotropic strain energy to account for anisotropic material having different mechanical properties in the given direction $\vec{a}$ as

$$\Delta w_{aniso} = \frac{\Delta\lambda}{2} I_1 I_4 + \Delta\mu I_5, \tag{15}$$

where $I_1 = trE$ was introduced before and $I_4 = \vec{a}^T E \vec{a}$ and $I_5 = \vec{a}^T E^2 \vec{a}$ are invariants of the strain tensor constructed with vector $\vec{a}$. Similar to the other invariants, I_4 and I_5 can also be expressed in terms of analogical invariants of Cauchy deformation tensor C, $I_4' = \vec{a}^T C \vec{a}$ and $I_5' = \vec{a}^T C^2 \vec{a}$

$$I_4 = \frac{1}{2}\vec{a}^T (C - I)\vec{a} = \frac{1}{2} I_4' - \frac{1}{2}, \tag{16}$$

$$I_5 = \frac{1}{4}\vec{a}^T (C^2 - 2C + I)\vec{a} = \frac{1}{4} I_5' - \frac{1}{2} I_4' + \frac{1}{2}, \tag{17}$$

2 Details of Triangular Biquadratic Springs implementation

2.1 Strain energy formalism in TRBS

All of the invariants necessary for evaluating the isotropic and anisotropic energy densities can be expressed in terms of deformation gradient tensor F. In the case of triangular elements, it is convenient to derive F in terms of position of the nodes in resting and deformed states of the element. The i'th shape

vector D_i corresponding with the node P_i in the resting shape (Figure 1B in the main text) is:

$$D_i = \frac{1}{A_P}(P_j - P_k)^{\perp} \quad ; \epsilon_{ijk} = 1, \tag{18}$$

where A_P is the resting area of the element, ϵ_{ijk} is the permutation symbol and $X^{\perp}$ is orthogonal to the vector X. In this case the expression for F becomes [2]

$$F = Q_i \otimes D_i, \tag{19}$$

where Q_i is the position vector of i'th node in the deformed shape.

Using these expressions, the Cauchy-Green deformation tensor becomes

$$C = (D_i \otimes D_j)(Q_i \cdot Q_j). \tag{20}$$

In the above equations and through out the text the repeated indices are summed over from 1 to 3, unless stated otherwise (we assume Einsteins summing convention).

These invariants can be calculated in terms of the angles α_i of resting shape, edges l_i, L_i of resting and deformed shapes and areas A_P, A_Q of resting and deformed shapes of triangular elements and the strain energy becomes [2]

$$w_{iso} = \frac{k_i}{4}(\Delta^2 l_i)^2 + \Sigma_{i \neq j} \frac{c_k}{2} \Delta^2 l_i \Delta^2 l_j, \tag{21}$$

where $\Delta^2 l_i = l_i^2 - L_i^2$ is the square elongation of the i'th edge and k_i and c_k are tensile and angular elasticities of TRBS given by:

$$k_i = \frac{2(\lambda + 2\mu)cot^2\alpha_i + 2\mu}{16A_P} = \frac{Y(2cot^2\alpha_i + 1 - \nu)}{16(1-\nu^2)A_P}, \tag{22}$$

and

$$c_k = \frac{24(\lambda + 2\mu)cot\alpha_i cot\alpha_j - 2\mu}{16A_P} = \frac{Y(2cot\alpha_i cot\alpha_j - 1 + \nu)}{16(1-\nu^2)A_P}. \tag{23}$$

By taking derivative of w respect to Q_i we can derive an expression for force f_i applied on the node i:

$$f_i = -A_P \left(\frac{\partial w_{iso}}{\partial Q_i} \right)^T = \Sigma_{j \neq i} k_k \Delta^2 l_k (Q_j - Q_i) + \Sigma_{j \neq i}(c_j \Delta^2 l_i + c_i \Delta^2 l_j)(Q_j - Q_i). \tag{24}$$

This expression provides the force on each node entirely in terms of node positions of the triangular elements in resting and deformed configurations.

2.2 Anisotropic TRBS implementation

In TRBS framework, Cauchy-Green deformation tensor invariants can be expressed in terms of position vectors Q_i and shape vectors D_i

$$I_1' = trC = (Q_i \cdot Q_j)(D_i \cdot D_j), \tag{25}$$

$$I_2' = trC^2 = (Q_m \cdot Q_n)(Q_r \cdot Q_s)(D_n \cdot D_r)(D_m \cdot D_s), \tag{26}$$

$$I_4' = \vec{a}^T C \vec{a} = (Q_m \cdot Q_n)(\vec{a} \cdot D_m)(\vec{a} \cdot D_n), \tag{27}$$

$$I_5' = \vec{a}^T C^2 \vec{a} = (Q_m \cdot Q_n)(Q_r \cdot Q_s)(D_n \cdot D_r)(\vec{a} \cdot D_m)(\vec{a} \cdot D_s), \tag{28}$$

where we continue using Einsteins summing convention.
The anisotropic correction term for the TRBS force on i'th node Δf_i can be derived from our definition of Δw_{aniso} (Equation 15) as

$$\Delta f_i = -A_P \frac{\partial \Delta w_{aniso}}{\partial Q_i} = -A_P \left[\frac{\Delta\lambda}{2} \left(I_4 \frac{\partial I_1}{\partial Q_i} + I_1 \frac{\partial I_4}{\partial Q_i} \right) + \Delta\mu \frac{\partial I_5}{\partial Q_i} \right]. \tag{29}$$

2.3 Strain and Stress in TRBS

The strain is a local measure of deformation. There exist different strain measures comparing the changes of relative material point positions between undeformed and deformed (current) configurations. We use two strain measures: Green-Lagrange strain E (Equation 4), which operates on undeformed (material) coordinates and Euler-Almansi strain e relating quantities in deformed (current) coordinates and is defined by:

$$e = \frac{1}{2}(I - F^{T^{-1}} F^{-1}) = \frac{1}{2}(I - b^{-1}), \tag{30}$$

where we introduced $b = FF^T$ as left CauchyGreen deformation tensor. The strain tensors are energy conjugates of appropriate stress measures. The second Piola-Kirchhoff stress tensor S is an energy

conjugate of Green-Lagrange strain E.

$$S = \frac{\partial W}{\partial E} \tag{31}$$

The Cauchy stress tensor σ is the energy conjugate of the Euler-Almansi strain e. The Cauchy stress tensor is related to second Piola-Kirchhoff stress by

$$\sigma = \frac{1}{det(F)} F S F^T. \tag{32}$$

In case of St. Venant-Kirchhoff material (1) these become:

$$S = \lambda(trE)I + 2\mu E \tag{33}$$

and

$$\begin{aligned} \sigma &= \frac{A_P}{A_Q}[FF^T(\lambda trE) + 2\mu F \frac{1}{2}(C - I)F^T] \\ &= \frac{A_P}{A_Q}\left[(\lambda trE - \mu)b + \mu b^2\right], \end{aligned} \tag{34}$$

where we used the fact that for TRBS $det(F) = \frac{A_Q}{A_P}$. Analogically the correction energy term from material anisotropy will give rise to stress correction terms

$$\begin{aligned} \Delta S &= \frac{\partial \Delta W}{\partial E}, \\ \Delta\sigma &= \frac{1}{det(F)} F \Delta S F^T. \end{aligned} \tag{35}$$

Direct calculation from anisotropic energy (15) gives

$$\begin{aligned} \Delta S &= \frac{\Delta\lambda}{2}(I_4 \frac{\partial I_1}{\partial E} + I_1 \frac{\partial I_4}{\partial E}) + \Delta\mu \frac{\partial I_5}{\partial E} \\ &= \frac{\Delta\lambda}{2}((a^T E a)I + (trE)(a \otimes a)) + \Delta\mu(E(a \otimes a) + (a \otimes a)E). \end{aligned} \tag{36}$$

Finally the expression for the Cauchy's stress including effects of anisotropy becomes

$$\sigma = \frac{A_P}{A_Q} F[S + \Delta S]F^T. \tag{37}$$

The total force $\vec{f}^{total}$ on the i'th node of each element can be calculated using the total second Piola-Kirchhoff stress $S + \Delta S$, deformation gradient tensor F and the corresponding shape vector D_i.

$$f_i^{total} = -A_P F(S + \Delta S) D_i. \tag{38}$$

3 Nonlinear Finite Element Method

The Finite Element Method (FEM) is well established procedure for solution of differential equations on complicated domains. In this paper we used a nonlinear formulation of shell FEM to check the effect of some simplistic assumptions of the TRBS on the behavior of our model. The detailed description of the nonlinear FEM used in this paper can be found in textbooks [3]. The derivation of the mechanical equilibrium in FEM is based on the weak formulation of elasticity equations - the principle of virtual work. The variation of the work δW due to the virtual rate of deformation tensor, $\delta\mathbf{d}$, and velocities, $\delta\mathbf{v}$, can be written in spatial or Eulerian form as

$$\delta W = \int_\omega \sigma : \delta\mathbf{d}\, dv - \int_\omega \mathbf{f}\cdot\delta\mathbf{v}\, dv - \int_{\partial\omega} \mathbf{t}\cdot\delta\mathbf{v}\, da = 0, \tag{39}$$

where σ is Cauchy's stress tensor, $\mathbf{f}$ and $\mathbf{t}$ are body forces and tractions respectively, ω and $\partial\omega$ denote the domain of interest and its boundary respectively and dv, da corresponding infinitesimal elements. The standard procedure of solving this problem is linearization and iterative steps with respect to trial deformation solution ϕ_k eg. by use of the Newton-Raphson method. The equilibrium equations linearized in the direction of increment $\mathbf{u}$ in ϕ_k can be written as:

$$D\delta W(\phi_k, \delta v)[\mathbf{u}] = D\delta W_{int}(\phi_k, \delta v)[\mathbf{u}] - D\delta W_{ext}(\phi_k, \delta v)[\mathbf{u}], \tag{40}$$

where

$$\delta W_{int}(\phi_k, \delta v)[\mathbf{u}] = \int_\omega \sigma : \delta\mathbf{d}\, dv \tag{41}$$

$$\delta W_{ext}(\phi_k, \delta v)[\mathbf{u}] = \int_\omega \mathbf{f}\cdot\delta\mathbf{v}\, dv + \int_{\partial\omega} \mathbf{t}\cdot\delta\mathbf{v}\, da, \tag{42}$$

are describing internal and external work components. A careful derivation shows that linearization of the internal work can be expressed as:

$$D\delta W_{int}(\phi_k, \delta v)[\mathbf{u}] = \int_\omega \delta \mathbf{d} : c : \epsilon \, dv + \int_\omega \sigma : [(\nabla \mathbf{u})^T \nabla \delta \mathbf{v}] \, dv. \tag{43}$$

Here $\delta\mathbf{d}$ is a virtual rate of deformation tensor, c is a fourth order spatial elasticity tensor, and ϵ is a small deformation strain tensor. The above equation constitutes a more general form of Equation 1, as here no specific assumptions about the material properties have been introduced. In our model we used the anisotropic material defined by strain energy density (Equations 8 and 15)

$$w = w_{iso} + \Delta w_{aniso}. \tag{44}$$

The Cauchy stress

$$\sigma = \frac{1}{det(F)} F : \frac{\partial w}{\partial E} : F^T \tag{45}$$

and elasticity tensor

$$c = \frac{1}{det(F)} F \otimes F : \frac{\partial^2 w}{\partial E^2} : F^T \otimes F^T \tag{46}$$

calculated for this strain energy density were used in solution of Equation 43.

Discretization of this equation will yield a stiffness matrix, which because of apparent symmetry of above equation in $\mathbf{u}$ and $\delta\mathbf{v}$ will be symmetric too. The discretization of $\mathbf{u}$ and $\delta\mathbf{v}$ is performed with respect to shape vectors, which provide a local support basis for the problem and which specific form depends on the choice of the finite element discretization.

3.0.1 Shell kinematics

We have used quadrilateral shell elements, which give a good description of thin curved surfaces and provide an alternative to TRBS. The shell description is essentially three-dimensional elasticity with specific kinematic and mechanical assumptions built in into the theory. Here we will in short present extensible director formulation of shell element kinematics which has been used for quadrilateral shells in the simulations. For details of shell kinematics see [4] and for specifics of extensible director approach see [5]. The undeformed geometry of shell is described in local shell coordinates ξ_i with respect to its

reference surface $\bar{\mathbf{X}}$ with the following relations:

$$\mathbf{X}(\xi_i) = \bar{\mathbf{X}}(\xi_\alpha) + \mathbf{G}(\xi_i) \tag{47}$$

$$\bar{\mathbf{X}}(\xi_\alpha) = \sum_{a=1}^{n} N_a(\xi_\alpha)\bar{\mathbf{X}}_a \tag{48}$$

$$\mathbf{G}(\xi_i) = \sum_{a=1}^{n} N_a(\xi_\alpha) z_a(\xi_3)\bar{\mathbf{G}}_a \tag{49}$$

$$z_a(\xi_3) = N_+(\xi_3) z_a^+ + N_-(\xi_3) z_a^- \tag{50}$$

$$N_+(\xi_3) = \frac{1}{2}(1+\xi_3), \quad N_-(\xi_3) = \frac{1}{2}(1-\xi_3), \tag{51}$$

where Latin and Greek indices are assumed to span from 1 to 3 and from 1 to 2 respectively. The unit vector $\bar{\mathbf{G}}$ is called the director and it describes position of the body particle with respect to the point on the reference surface $\bar{\mathbf{X}}$. This point in turn is described by the two dimensional shape functions N_a and nodal points of the element $\bar{\mathbf{X}}_a$. The function z_a describes the thickness of the element in terms of the distance from the reference surface to the bottom, z_a^-, and top, z_a^+, surfaces. The similar interpolation is used to describe the current configuration of the shell element and in consequence the displacement vectors. The director vector in deformed configuration is no longer required to be of unit length which takes into account thickness changes. The definitions of the shape functions (and their respective derivatives) allow to parametrize the deformations and deformation gradient tensors with nodal positions of elements in current and unreformed configurations. These quantities together with the definitions of appropriate material provide the way to calculate the strain, stress and elasticity tensors.

References

1. Mase GT, Smelser RE, Mase GE (2010) Continuum Mechanics For Engineers. CRC Press, 370 pp.

2. Delingette H (2008) Triangular springs for modeling nonlinear membranes. IEEE Transactions on Visualization and Computer Graphics 14: 329–341.

3. Bonet J, Wood RD (1997) Nonlinear continuum mechanics for finite element analysis. Cambridge University Press.

4. Hughes TJR (2000) The Finite Element Method: Linear Static and Dynamic Finite Element Analysis. Dover Publications, 672 pp.

5. Simo J (1989) On a stress resultant geometrically exact shell model. Part I: Formulation and optimal parametrization. Computer Methods in Applied Mechanics and Engineering 72: 267–304.
